# Therapeutic Effects of 25-Hydroxyvitamin D on the Pathological Process of Benign Prostatic Hyperplasia: An In Vitro Evidence

**DOI:** 10.1155/2021/4029470

**Published:** 2021-10-11

**Authors:** Yanbo Chen, Hui Xu, Chong Liu, Meng Gu, Qi Chen, Ming Zhan, Zhong Wang

**Affiliations:** ^1^Department of Urology, Shanghai Ninth People's Hospital Affiliated to Shanghai Jiaotong University School of Medicine, Shanghai, China; ^2^Department of Emergency, Shanghai Ninth People's Hospital Affiliated to Shanghai Jiaotong University School of Medicine, Shanghai, China

## Abstract

The pathogenesis of benign prostatic hyperplasia (BPH) is extremely complicated which involving the multiple signaling pathways. The deficiency of vitamin D is an important risk factor for BPH, and exogenous vitamin D is effective for the treatment of BPH. In this study, we provided in vitro mechanical evidence of vitamin D as a treatment for BPH using BPH-1, WPMY-1, and PBMC cells. We found that 25-hydroxyvitamin D (25-OH D) level is decreased in BPH and closely correlated with age, prostate volume, maximum flow, international prostate symptom score, and prostate-specific antigen of the BPH patients. We further revealed that 25-OH D ameliorated TGF-*β*1 induces epithelial-mesenchymal transition (EMT) of BPH-1 cells and proliferation of WPMY-1 cells via blocking TGF-*β* signaling. Moreover, 25-OH D was able to block NF-*κ*B signaling in PBMCs of BPH patients and STAT3 signaling in BPH cells to relieve inflammation. 25-OH D also protects BPH cells from inflammatory cytokines selected by PBMCs. Finally, we uncovered that 25-OH D alleviated prostate cell oxidative stress by triggering Nrf2 signaling. In conclusion, our data verified that 25-OH D regulated multiple singling pathways to restrain prostate cell EMT, proliferation, inflammation, and oxidative stress. Our study provides in vitro mechanical evidence to support clinical use of vitamin D as a treatment for BPH.

## 1. Introduction

Benign prostatic hyperplasia (BPH) is a chronic progressive condition which impacts a substantial number of older men [1, 2]. With aging of the world population, the incidence rate of BPH has increased rapidly [3]. BPH is now a big health problem, which not only influences the life quality of patients but also brings heavy burden to society [4].

Many risk factors including, aging, inflammation, hormones, oxidative stress, physical activity, and dietary factors are considered to participate in BPH development, leading to the pathogenesis of BPH extremely multifactorial [5]. Many studies and our previous research have illustrated that the activated epithelial-mesenchymal transition (EMT) process of prostate epithelial cells and the incontrollable growth of prostate stromal cells are essential pathogenesis processes of BPH; in this progression, excessive levels of transforming growth factor-beta 1(TGF-*β*1) and then activated TGF-*β*/Smad signaling are important initiators [6–8]. Otherwise, abnormal activation of some inflammatory signaling, such as NF-*κ*B and STAT3 signaling pathways, leads to abnormal secretion of inflammatory cytokines, which is an important pathophysiological basis for chronic inflammatory changes of prostate [9, 10]. According to many studies, oxidative stress is also considered to play a role in the development of BPH, in which Nrf2 signaling is deficient and insufficient for antioxidant response [11, 12]. Therefore, interfering the activation of these signaling may produce therapeutic effects on the pathogenesis of BPH.

Except for the well-known function in calcium metabolism, vitamin D also helps to prevent the occurrence and development of many chronic diseases, including cardiovascular diseases, diabetes, and malignant tumors [13]. Accumulating evidence indicates that low vitamin D, especially the active 25-hydroxyvitamin D (25-OH D), is deficient in BPH patients and may be closely associated with the disease pathophysiologic processes [14, 15]. More importantly, a recent randomized controlled trial uncovered that vitamin D supplementation is effective in reducing prostate volume and PSA levels, as well as improving BPH symptoms [16]. However, there has been no enough in vitro evidence to clarify the mechanisms of 25-OH D in reversing the BPH pathological process to support the findings of the in vivo data. Therefore, in this study, we want to conduct in vitro experiments to further investigate the mechanism of vitamin D as a treatment for BPH.

## 2. Materials and Methods

### 2.1. Patients

A total of 160 patients diagnosed with BPH and 120 age-matched healthy individuals were recruited at the Shanghai Ninth People's Hospital. The inclusion criteria were signed the approved informed consent and clinically diagnosed as BPH. The exclusion criteria were history of other acute or chronic diseases, history of other urinary diseases and surgery, and use of any medications that may influence the vitamin D level (corticosteroids, phenytoin, phenobarbital, vitamin D supplements, or others) over the past three months. This study was approved by the Ethical Committee of Shanghai Ninth People's Hospital, and written informed consents were obtained from all participants. Serum 25-OH D level was measured using electrochemiluminescence immunoassay. All participants underwent digital rectal examination and transrectal ultrasound examination. Prostate volume was calculated by ultrasound using the ellipsoid method [17].

### 2.2. Cell Culture, Treatment, and Transfection

Benign prostatic hyperplasia epithelial-1 (BPH-1) cells were acquired from German Collection of Microorganisms and Cell cultures (Leibniz Institute DSMZ, Germany) and maintained in RPM-1640 medium (Gibco, USA) supplemented with 10% FBS (Gibco). SV40 large-T antigen-immortalized stromal cell line WPMY-1 was acquired from the Stem Cell Bank, Chinese Academy of Sciences (Shanghai, China) and maintained in DMEM medium (Gibco supplemented with 10% FBS). Peripheral blood mononuclear cells (PBMCs) were separated from peripheral venous blood of BPH patient using Ficoll-Paque reagent (Sigma) and density gradient centrifugation method. PBMCs were maintained in RPM-1640 medium (Gibco) supplemented with 10% FBS and activated with Leukocyte Activation Cocktail (BD Pharmingen, USA). Cells were treated with 5 ng/ml TGF-*β*1 (RD Biosciences, USA) or 70 ng/ml 25-hydroxyvitamin D3 (Sigma-Aldrich, USA) for 72 h. Transfection of BPH-1 and WPMY-1 cells was performed using Lipofectamine 3000 reagent (Invitrogen, USA), and transfection of PBMCs was performed using Cell Line Nucleofector Kit (Lonza, Switzerland).

### 2.3. Quantitative Real-Time PCR (qRT-PCR)

TRIzol (Invitrogen) was used to extract total RNAs from cells, and PrimeScript RT reagent kit (Takara, Japan) was used to reversely transcribe RNAs into cDNAs. A SYBR real-time PCR kit (Takara) was applied to perform qPCR assay. The qPCR reaction was run on thermocycling conditions: 95°C for 2 min, followed by 40 cycles of 95°C for 20 sec, 60°C for 30 sec, and 72°C for 20 sec. Relative mRNA expressions were normalized to GAPDH expression and calculated by the 2^-*ΔΔ*Ct^ method. The sequences of the primers in the qRT-PCR assays are *α*-smooth muscle actin (*α*-SMA), forward, CTATGAGGGCTATGCCTTGCC, and reverse, GCTCAGCAGTAGTAACGAAGGA; N-cadherin: forward, AGCCAACCTTAACTGAGGAGT, and reverse, GGCAAGTTGATTGGAGGGATG; E-cadherin: forward, CGAGAGCTACACGTTCACGG, and reverse, GGGTGTCGAGGGAAAAATAGG; CCND1: forward, CTGGAGGTCTGCGAGGAACA, and reverse, CTTAGAGGCCACGAACATGCA; p21: forward, CGATGGAACTTCGACTTTGTCA, and reverse, GCACAAGGGTACAAGACAGTG; Nfr2, forward, TCCAGTCAGAAACCAGTGGAT, and reverse, GAATGTCTGCGCCAAAAGCTG.

### 2.4. Protein Extraction and Western Blot Assay

RIPA lysis buffer was applied to isolate total proteins from cells. Proteins were then separated by SDS-PAGE and electrophoretically transferred onto PVDF membranes (Millipore). After blocked with 5% skim milk, the membranes were incubated with primary antibodies overnight at 4°C. After washing, the membranes were then incubated with secondary antibodies at room temperature for 2 h. Antibodies used for the immunoreactivity were Smad2 [1 : 1000, #5339, Cell Signaling Technology (CST), USA]; Phospho-Smad2 (Ser465/Ser467) (1 : 1000, #18338, CST); Smad3 (1 : 1000, #9523, CST); Phospho-Smad3 (Ser423/425) (1 : 1000, #9520, CST); *α*-SMA (1 : 1000, #19245, CST); N-cadherin (1 : 1000, #13116, CST); E-cadherin (1 : 50, ab1416, Abcam, USA); CCND1 (1 : 200, ab16663, Abcam); p21 (1 : 1000, ab188224, Abcam); phospho-NF-*κ*B p65 (Ser536) (1 : 1000, #3033, CST); NF-*κ*B p65 (1 : 1000, #8242, CST); phospho-Stat3 (Tyr705) (1 : 2000, #9145, CST); Stat3 (1 : 1000, #9139, CST); Nfr2 (1 : 1000, #12721, CST); and GAPDH (1 : 2500, ab9485, Abcam).

### 2.5. Luciferase Assay

The luciferase assay was conducted to assess the transcriptional activity of Smad2/Smad3/Smad4, NF-*κ*B p65, STAT3, and Nfr2. Luciferase reporters containing Smad2/Smad3/Smad4, NF-*κ*B p65, STAT3, or Nfr2 transcription complex binding promoter elements were synthesized by Hanyin Biotechnology (Shanghai, China). After different treatments, the above reporters were transfected into cells along with pRL-TK vector (Promega, USA). Luciferase activities were detected by a Dual-Luciferase reporter assay system (Promega) to represent the relative transcriptional activity of the above transcription factors.

### 2.6. EMT Evaluation

The evaluation of EMT was the same as described before [8]. A phase-contrast microscope was used to observe elongated fibroblast-like morphology with scattered distribution of BPH-1 cells. At the same time, qRT-PCR and western blot assays were applied to detect the expressions of EMT biomarkers including *α*-SMA, N-cadherin, and E-cadherin.

### 2.7. Cell Proliferation Assay

CCK-8 assay was used to evaluate cell proliferation vitality. WPMY-1 cells (1 × 10^4^) were seeded into 96-well plates for 24 h before reagents treatment. After 24, 48, and 72 hours, cells were incubated with CCK-8 reagents (MedChemExpress) in a cell culture incubator for 1 hour. A microplate reader (Bio-Rad) was used to measure the absorbance at a wavelength of 450 nm. At the same time, qRT-PCR and western blot assays were applied to detect the expressions of cell cycle-related genes, including CCND1 and p21.

### 2.8. Cytokine Detection

Transcriptional levels of inflammatory cytokines including IL-6, IL-23, and IL-17 were analyzed by qRT-PCR. Protein levels in cell supernatant were detected using ELISA assays with ELISA kits (ab178013, ab221837, ab100556, Abcam). At the same time, luciferase assays were applied to analyze the activation of inflammation signaling pathways including NF-*κ*B and STAT3 pathway.

### 2.9. Oxidative Stress Assessment

MDA level and reactive oxygen species (ROS) were detected to assess oxidative stress. A Lipid Peroxidation MDA Assay Kit (S0131, Beyotime, China) was used to analyze MDA level, and a Reactive Oxygen Species Assay Kit (S0033, Beyotime) was used to measure ROS level. At the same time, luciferase assays were applied to analyze the activation of the Nfr2 oxidative stress signaling pathway.

### 2.10. Statistical Analysis

Statistical analysis was carried out using SPSS 18.0 software (SPSS, IL, USA). All data from at least three independent experiments were presented as the mean ± standard deviation (SD). Student's *t* test or one-way analysis of variance followed by Dunnett-*t* test was performed to assess comparison across groups. *P* value < 0.05 was considered to be statistically significant.

## 3. Results

### 3.1. 25-OH D Level Is Decreased in BPH and Associated with Various Clinical Parameters

Of the 160 BPH patients, the medium serum level of 25-OH D was significantly lower than that of the 120 age-matched healthy individuals (16.73 ng/mL vs. 35.28 ng/mL, *P* < 0.001). One hundred and three (64.4%) BPH patients were identified with 25-OH D deficiency (<20 ng/mL). 25-OH D deficiency was closely correlated with age, prostate volume, maximum flow, international prostate symptom score (IPSS), and prostate-specific antigen (PSA) of the BPH patients (Table 1).

### 3.2. 25-OH D Ameliorates TGF-*β*1 Induces EMT of BPH-1 Cells and Proliferation of WPMY-1 Cells via Blocking TGF-*β* Signaling

**Figure 1:**
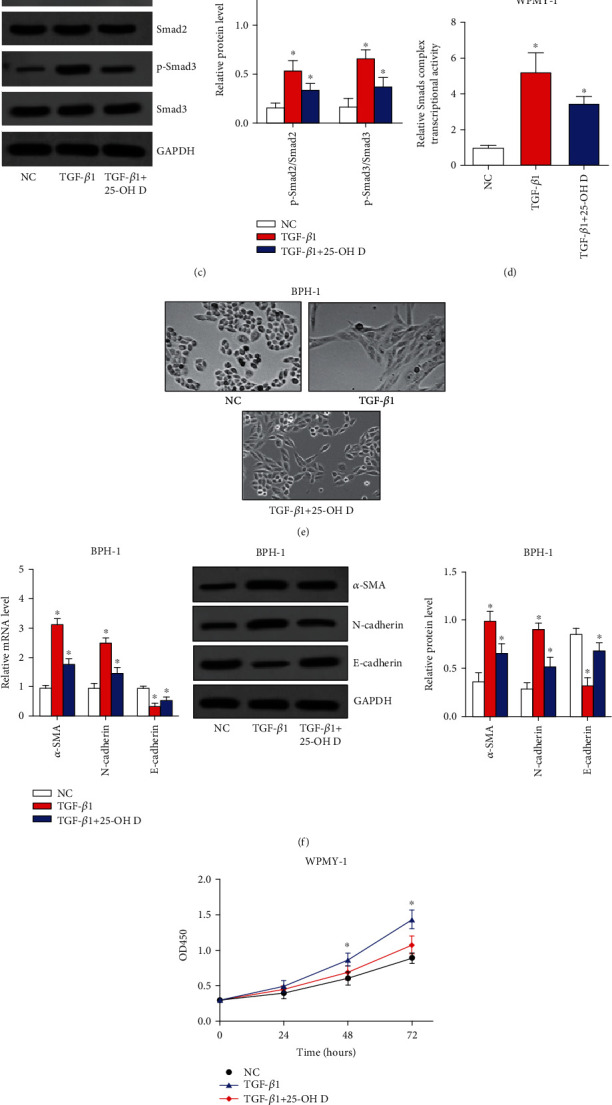
25-OH D ameliorates TGF-*β*1 induces EMT of BPH-1 cells and proliferation of WPMY-1 cells via blocking TGF-*β* signaling. (a) Phosphorylation of Smad2 and Smad3 was detected by western blot assay after BPH-1 cells were treated with TGF-*β*1 and 25-OH D. (b) Transcriptional activity of Smad2/Smad3/Smad4 complex was analyzed by luciferase assay after BPH-1 cells were treated with TGF-*β*1 and 25-OH D. (c) Phosphorylation of Smad2 and Smad3 was detected by western blot assay after WPMY-1 cells were treated with TGF-*β*1 and 25-OH D. (d) transcriptional activity of Smad2/Smad3/Smad4 complex was analyzed by luciferase assay after WPMY-1 cells were treated with TGF-*β*1 and 25-OH D. (e) EMT of BPH-1 cells were evaluated by observing elongated fibroblast-like morphology with scattered distribution. (f) The mRNA and protein levels of EMT biomarkers including *α*-SMA, E-cadherin, and N-cadherin were detected by qRT-PCR and western blot assays. (g) Proliferation ability of WPMY-1 cells was assessed by CCK8 assays. (h) The mRNA and protein expressions of cell cycle-related genes including CCND1 and p21 were analyzed by qRT-PCR and western blot assays. ^∗^*P* < 0.05.

For activated TGF-*β*/Smad signaling is an important pathophysiological basis of BPH, first, we treated BPH-1 cells and WPMY-1 cells exposed to TGF-*β*1 with 25-OH D. We revealed that 25-OH D could significantly suppress the phosphorylation of Smad2 and Smad3, and thereby restrained the transcriptional activity of Smad2/Smad3/Smad4 complex induced by TGF-*β*1 in both BPH-1 cells and WPMY-1 cells (Figures 1(a)–1(d)). As a result, 25-OH D obviously reversed the EMT process of BPH-1 cells result from the TGF-*β*1 treatment (Figure 1(e)). Accordingly, 25-OH D also attenuated the expression changes of EMT biomarkers including *α*-SMA, N-cadherin, and E-cadherin caused by TGF-*β*1 (Figure 1(f)). Also, in WPMY-1 cells, 25-OH D obviously reversed cell proliferation (Figure 1(g)) and expression changes of cell cycle-related genes including CCND1 and p21 caused by TGF-*β*1 (Figure 1(h)). Taken together, these data suggested that 25-OH D-ameliorated TGF-*β*1 induces EMT of BPH-1 cells and proliferation of WPMY-1 cells via blocking TGF-*β* signaling.

### 3.3. 25-OH D Relieves Inflammation of BPH via Blocking NF-*κ*B and STAT3 Signaling Pathways

**Figure 2:**
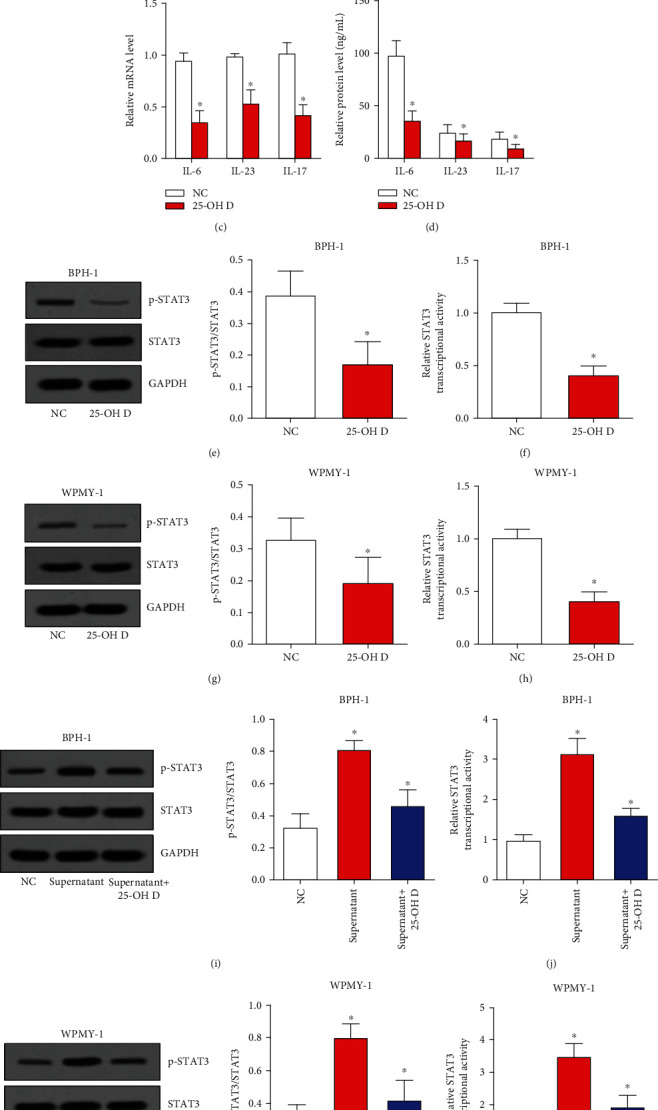
25-OH D relieves inflammation of BPH via blocking NF-*κ*B and STAT3 signaling pathways. (a) Phosphorylation of NF-*κ*B p65 was detected by western blot assay after PBMCs were treated with 25-OH D. (b) Transcriptional activity of NF-*κ*B p65 was analyzed by luciferase assay after PBMCs were treated with 25-OH D. (c) and (d) The mRNA and protein levels of inflammatory cytokines were detected by qRT-PCR and ELISA assays. (e)–(h) Phosphorylation and transcriptional activity of STAT3 were analyzed by western blot and luciferase assay after BPH-1 (e) and (f) or WPMY-1 (g) and (h) cells were treated with 25-OH D. (e)–(h) Phosphorylation and transcriptional activity of STAT3 were analyzed by western blot and luciferase assay after BPH-1 (i) and (j) or WPMY-1 (k) and (l) cells were treated with supernatant of PBMCs and 25-OH D. ^∗^*P* < 0.05.

Inflammation is another pathophysiological basis for chronic changes of BPH, and we then investigated the roles of 25-OH D on inflammation. Our data suggested that the phosphorylation as well as the transcriptional activity of NF-*κ*B p65 in PBMCs of BPH patients was significantly restrained by 25-OH D treatment (Figures 2(a) and 2(b)). Moreover, the transcriptional level and protein level in cell supernatant of inflammatory cytokines downstream of NF-*κ*B including IL-6, IL-23, and IL-17 were reduced by 25-OH D treatment (Figures 2(c) and 2(d)). In BPH-1 cells and WPMY-1 cells, 25-OH D could decrease the phosphorylation and transcriptional activity of STAT3 (Figures 2(e)–2(h)). Subsequently, the cell supernatant of PBMCs without 25-OH D treatment was used to treat BPH-1 cells and WPMY-1 cells. The results showed that the phosphorylation as well as the transcriptional activity of STAT3 in both BPH-1 cells and WPMY-1 cells was significantly upregulated by cell supernatant of PBMCs and then attenuated by 25-OH D (Figures 2(i)–2(l)). In summary, these results indicated that 25-OH D was able to block NF-*κ*B signaling in PBMCs of BPH patients and STAT3 signaling in BPH cells to relieve inflammation. 25-OH D also protects BPH cells from inflammatory cytokines selected by PBMCs.

### 3.4. 25-OH D Alleviates Prostate Cell Oxidative Stress by Triggering Nrf2 Signaling

**Figure 3:**
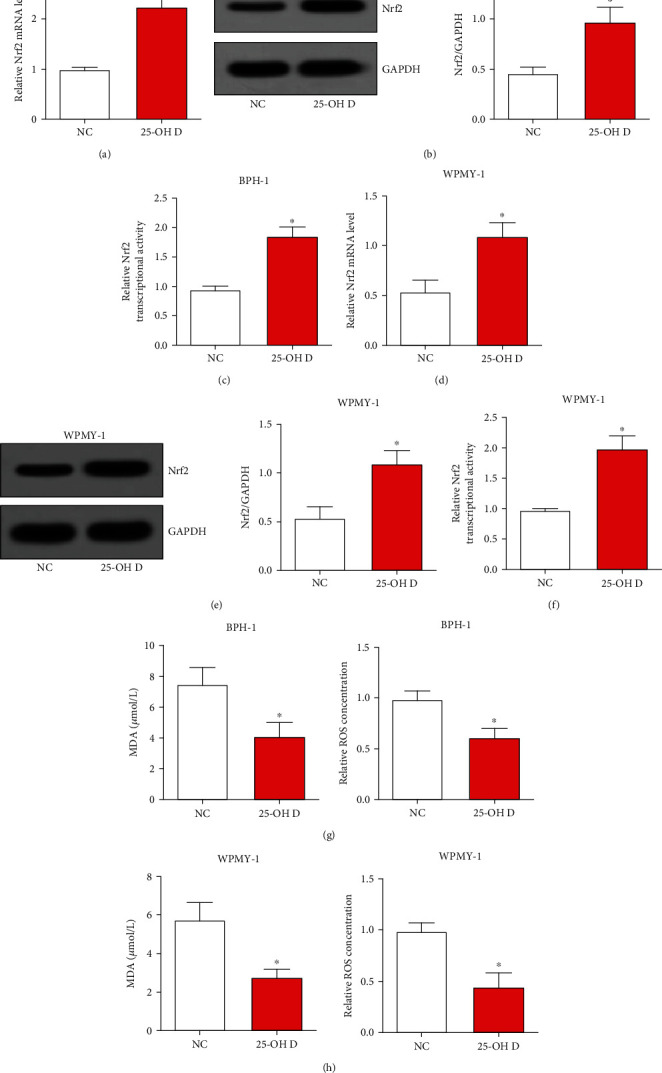
25-OH D alleviates prostate cells oxidative stress by triggering Nrf2 signaling. (a) and (b) The mRNA and protein levels of Nrf2 were analyzed by qRT-PCR and western blot assays after BPH-1 cells were treated with 25-OH D. (c) Transcriptional activity of Nrf2 was assessed by luciferase assay after BPH-1 cells were treated with 25-OH D. (d) and (e) The mRNA and protein levels of Nrf2 were analyzed by qRT-PCR and western blot assays after WPMY-1 cells were treated with 25-OH D. (f) Transcriptional activity of Nrf2 was assessed by luciferase assay after WPMY-1 cells were treated with 25-OH D. (g) and (h) The MDA level and relative ROS level were detected after 25-OH D treatment in both BPH-1 cells and WPMY-1 cells. ^∗^*P* < 0.05.

As oxidative stress plays a role in the development of BPH, we then investigated the molecular function of 25-OH D in regulating oxidative stress. Our data showed that 25-OH D significantly upregulated Nrf2 expression and transcriptional activity of Nrf2 in BPH-1 cells and WPMY-1 cells (Figures 3(a)–3(f)). The MDA level and relative ROS level were significantly decreased after 25-OH D treatment in both BPH-1 cells and WPMY-1 cells (Figures 3(g) and 3(h)). Because Nrf2 signaling is an antioxidant pathway, we concluded that 25-OH D alleviated prostate cell oxidative stress by triggering Nrf2 signaling.

## 4. Discussion

BPH occurs when both stromal and epithelial cells of the prostate in the transitional zone proliferate, causing prostate enlargement [18]. The pathogenesis of BPH is multifactorial and largely unknown, and several mechanisms seem to be involved in the initiation and development. Therefore, it is vital important to find out the pathogenic mechanisms of BPH, so as to develop effective treatments based on these mechanisms.

Studies have demonstrated the deficiency of vitamin D is an important risk factor of BPH, and exogenous vitamin D is effective for the treatment of BPH [14–16]. Our study is the first to provide in vitro evidence to support these clinical findings in vivo. In this study, the roles of vitamin D in reversing the pathological process of BPH are illustrated in multiple biological processes including EMT, cell proliferation, inflammation, and oxidative stress. This study was conducted in vitro using BPH-1 and WPMY-1 cells which were also used in our previous study [8].

Many studies and our previous research have illustrated that excessive level of TGF-*β*1 and then activated TGF-*β*/Smad signaling are important initiators for the EMT process of prostate epithelial cells and the incontrollable growth of prostate stromal cells [6–8]. Some studies have also confirmed that vitamin D suppressed TGF-*β* signaling to play therapeutic effects in some diseases both in vitro and in vivo [19–21]. Here, we showed that 25-OH D was able to ameliorate TGF-*β*1 induces EMT of BPH-1 cells and proliferation of WPMY-1 cells via blocking TGF-*β* signaling. So, we think these in vitro data suggest that 25-OH D may inhibit fibrosis and growth of the prostate in the process of BPH.

In addition, inflammation is another risk factor that contributed to the occurrence and development of BPH [9, 10]. This progression involves the abnormal activation of some inflammatory signaling, such as NF-*κ*B and STAT3 signaling pathways. In many other diseases, but not in BPH, vitamin D has already been demonstrated to play anti-inflammatory roles by deactivating NF-*κ*B and STAT3 signaling pathways [22–25]. In this study, our results indicated that 25-OH D was able to block NF-*κ*B signaling in PBMCs of BPH patients to reduce the secretion of inflammatory cytokines in serum. On the other hand, 25-OH D could inhibit STAT3 signaling in BPH cells to relieve inflammation and also protect BPH cells from inflammatory cytokines selected by PBMCs. In summary, these in vitro data indicated that 25-OH D may play anti-inflammatory roles in the process of BPH.

**Figure 4:**
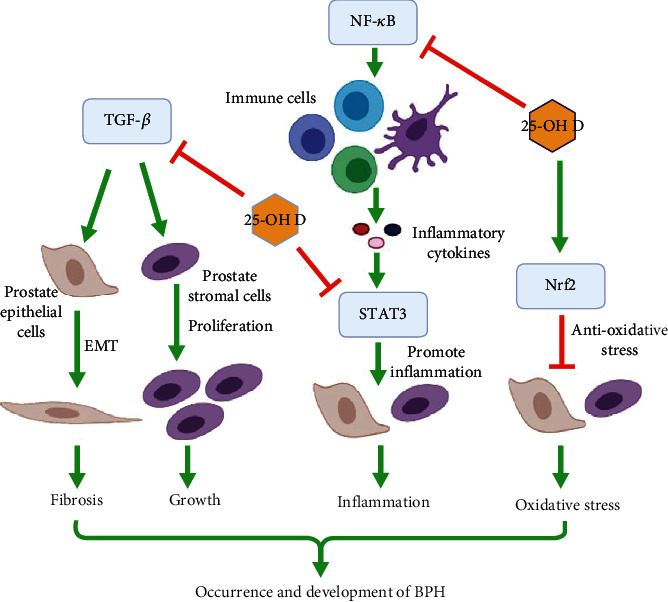
Diagram showing the roles of 25-OH D in regulating TGF-*β*, NF-*κ*B, STAT3, and Nrf2 singling pathways in BPH.

Otherwise, oxidative stress has been reported to play a role in the progression of BPH [11, 12]. Nrf2 signaling is an important endogenous antioxidant stress pathway, which is deficient and insufficient for antioxidant response in BPH [11, 12]. According to many studies, vitamin D could raise Nrf2 expression and activate Nrf2 signaling to reduce oxidative stress in human diseases [26–28]. The present study revealed that 25-OH D was able to alleviate prostate cell oxidative stress by triggering Nrf2 signaling, which indicated a potential antioxidative stress role of 25-OH D in the process of BPH. To sum up the above discussion, we drew a diagram to show the roles of 25-OH D in regulating these signaling pathways in BPH (Figure 4).

Although there has been a randomized controlled trial using vitamin D supplementation in the treatment of BPH symptoms [16], some animal experiments may make our in vitro data more convincing. This is a limitation of the present study.

In conclusion, our data verified that 25-OH D blocked TGF-*β* signaling, NF-*κ*B signaling, STAT3 signaling, and activated Nrf2 singling to restrain prostate cell EMT, proliferation, inflammation, and oxidative stress. Our study provides in vitro mechanical evidence to support clinical use of vitamin D as a treatment for BPH.

## Figures and Tables

**Table 1:** Comparison of various parameters in BPH patients with 25-OH D deficiency and insufficiency or normal.

Parameters	25-OH D		*P* value
	Deficiency	Insufficiency or normal	
*N*	103	57	—
Age (year)	73 (66–79)	67 (58–73)	0.023
Body mass index (kg/m^2^)	23.25 (17.31–38.72)	22.65 (16.98–38.52)	0.638
Prostate volume (mL)	46.5 (32.6-57.3)	31.4 (23.6-37.3)	<0.001
Maximum flow (mL/s)	14.44 (9.11-19.65)	21.37 (13.63-31.25)	<0.001
IPSS	4.31 (2.86-6.14)	1.79 (1.12-3.32)	<0.001
Serum PSA (ng/mL)	3.02 (2.53-4.61)	2.13 (1.86-3.36)	<0.001
Serum testosterone (ng/mL)	4.56 (4.09-6.23)	4.73 (4.15-6.42)	0.412
Serum SHBG (nmol/L)	41.32 (26.59-60.47)	41.89 (27.12-63.83)	0.321
Serum albumin (mg/dL)	4.52 (3.95-4.76)	4.65 (3.89-4.86)	0.614

Data were presented as medium (interquartile range). Shapiro-Wilk test was used to validate the nonnormal distribution, and Levene test was used for equality of variance. Mann–Whitney *U* test was applied for comparison of quantitative variables. IPSS: International Prostate Symptom Score; PSA: prostate-specific antigen; SHBG: sex hormone-binding globulin.

## Data Availability

The data used to support the findings of this study are included within the article.
